# *Porphyromonas gingivalis* may interfere with conception in women

**DOI:** 10.1080/20002297.2017.1330644

**Published:** 2017-06-12

**Authors:** Susanna Paju, Juha Oittinen, Henna Haapala, Sirkka Asikainen, Jorma Paavonen, Pirkko J. Pussinen

**Affiliations:** ^a^ Oral and Maxillofacial Diseases, Faculty of Medicine, University of Helsinki and Helsinki University Hospital, Helsinki, Finland; ^b^ Faculty of Dentistry, Health Sciences Center, Kuwait University, Safat, Kuwait; ^c^ Obstetrics and Gynecology, Faculty of Medicine, University of Helsinki and Helsinki University Hospital, Helsinki, Finland

**Keywords:** Periodontal-systemic disease interactions, microbiology, oral hygiene, periodontal diseases, periodontitis, women’s health

## Abstract

In this observational and prospective study, we investigated if microbiological and serological markers of periodontitis associated with conception in 256 non-pregnant women (*Mage* = 29.2 years; range 19–42 years). Clinical oral and gynecological examinations were performed, major periodontal pathogens in the saliva were detected, and serum and saliva antibodies against major periodontal pathogens were analyzed. The follow-up period for becoming pregnant was 12 months. *Porphyromonas gingivalis* was significantly (*p* = 0.032) more frequently detected in the saliva among those who did not become pregnant (8.3%) than among those who became pregnant (2.1%). The median levels of salivary P. gingivalis immunoglobulin A (IgA; p = 0.006) and IgG (*p* = 0.007) antibodies were higher among those who did not become pregnant compared to those who became pregnant. Hazard ratios (HR) for not becoming pregnant were HR = 3.75 (95% confidence interval [CI] 1.01–13.9; p = 0.048) if the subject was polymerase chain reaction–positive for *P. gingivalis* with high salivary antibodies against it, and HR = 1.62 (95% CI 1.03–2.54; *p* = 0.035) if she had high levels of serum P. gingivalis IgA and signs of periodontal infection. *P. gingivalis* associated with no success in getting pregnant.

## Introduction

Periodontal diseases are prevalent worldwide. Severe chronic periodontitis is the sixth most common medical condition according to the Global Burden of Disease Study [1]. Periodontal infection has been linked to several medical conditions such as cardiovascular diseases, diabetes, respiratory infections, and adverse pregnancy outcomes, including premature birth and low birth weight [2,3]. This systemic connection may be mediated by bacterial lipopolysaccharide (LPS), resulting in endotoxemia and a subsequent low-grade pro-inflammatory state [4]. Gram-negative bacteria, such as *Porphyromonas gingivalis* and *Aggregatibacter actinomycetemcomitans*, that are enriched in the biofilm of periodontal infection may contribute to endotoxemia [5,6] and therefore contribute to systemic inflammation. These species may also give rise to molecular mimicry, that is, cross-activation of autoreactive immune cells by pathogen-associated epitopes, which have harmful implications [7,8].

Several causes, such as ovulation disorders, ovarian insufficiency, Fallopian tube pathology, endometriosis, and uterine or cervical abnormalities, may affect fertility in women. Infection and inflammation play a role in infertility by affecting the ovary, uterus, the embryo, and implantation, and inflammation may interfere with ovulation and hormone production as well as contributing to endometriosis [9]. An association between periodontitis and endometriosis has previously been suggested [10]. Periodontitis and infertility share common risk factors such as age, obesity, and tobacco smoking, but the association still needs clarification.

Data on the influence of periodontitis on conception or the success of becoming pregnant are limited. Clinical periodontitis was associated with delayed conception among non-Caucasians, but because similar results were not found in Caucasians, it is unknown whether this was related to periodontitis or confounding factors [11]. Women with poor clinical periodontal status and signs of gingival inflammation seem to have worse outcomes by *in vitro* fertilization [12]. No data on the effect of microbiological or serological markers of periodontal infection on conception are available.

An observational study was conducted in a prospective setting with a cohort of young women who were hoping to become pregnant. The goal was to study whether microbiological or serological markers of common periodontal pathogens are associated with conception in women.

## Material and methods

### Study population

The study sample comprised 256 healthy non-pregnant Caucasian women (*M*_age_ = 29.2 years; range 19–42 years) [13]. Subjects were enrolled from the general community from the Helsinki University Central Hospital area in Southern Finland by newspaper advertisements. Inclusion criteria were that they were not pregnant, were menstruating regularly, and had discontinued contraception in order to become pregnant. Exclusion criteria included a history of preterm delivery and use of antibiotics within the preceding 2 weeks. All the 256 entering subjects also completed the study. The study was approved by the Ethics Committee of the Department of Obstetrics and Gynecology, University of Helsinki, Finland. All the subjects provided written informed consent. This study conforms to STROBE guidelines. The sample size was calculated with 80% power and at 5% significance level to find 20% difference in the serum antibody levels between women with different conception outcomes.

### Baseline examinations and follow-up

Subjects were interviewed on their medical history, tobacco smoking, oral hygiene habits, previous dental visits, and socioeconomic status. Social class was determined according to the woman’s education level and divided into three categories: low – junior high school or vocational school; medium – college, polytechnic, or a higher vocational institute; or high – academic university. Clinical oral and gynecological examinations were performed, as previously described [13]. Briefly, the oral examination measured the presence of caries lesions and periodontal disease (periodontal pocket depth; visible plaque; gingival bleeding on probing; and clinical periodontal attachment loss [PAL]), and stimulated saliva was collected by chewing paraffin for 2 min. A periodontal pocket depth of ≥4 mm was considered as a pocket, and clinical attachment loss of ≥1 mm was considered as PAL. Gynecological speculum examination was performed, and a vaginal swab was taken for the diagnosis of bacterial vaginosis (BV). The results of the clinical oral and gynecological examinations were disseminated to participants immediately after the examinations.

Saliva samples were processed for polymerase chain reaction (PCR) analysis to detect two major periodontal pathogens – *P. gingivalis* and *A. actinomycetemcomitans* – using species-specific primers, as previously described [14]. Saliva and serum samples were analysed for immunoglobululin G (IgG) and IgA antibodies against *P. gingivalis* and *A. actinomycetemcomitans* by an enzyme-linked immunosorbent assay (ELISA) with a mixture of three *P. gingivalis* serotypes and six *A. actinomycetemcomitans* serotypes, as previously described [15]. The antibody levels are presented as absorbance (AU) and ELISA units (EU) for saliva and serum, respectively. Threshold for seropositivity was determined as ≥2.0 EU for IgA and ≥5.0 EU for IgG, according to Pussinen et al. [15].

Vaginal samples were Gram-stained for the diagnosis of BV based on Nugent’s criteria [16]. Study subjects were followed to establish whether they did (*n* = 205) or did not (*n* = 51) become pregnant during the observation period of 12 months.

### Statistical analysis

A chi-square test was used to calculate the frequency distributions, and a non-parametric Mann–Whitney *U*-test was used to calculate the differences in continuous variables between conception outcomes. The correlations between serum and saliva antibody levels were analysed with Spearman’s correlation coefficient. The Cox regression models were adjusted for age, current smoking, socioeconomic status, BV, previous deliveries, and clinical PAL. The goodness of fit was ascertained by a Hosmer–Lemeshow test. The hazard ratios (HR) with corresponding 95% confidence intervals (CI) are presented for the association between conception outcomes and measured parameters. A *p*-value of <0.05 was considered to be statistically significant. IBM SPSS Statistics for Windows v22 (IBM Corp., Armonk, NY) was used for all calculations.

## Results

Table 1 shows selected characteristics of the study subjects. Those who did not become pregnant were older, more frequently current smokers, and had higher median C-reactive protein (CRP) concentrations compared to those who became pregnant.

**Table 1.** Baseline characteristics of study subjects (*n* = 256) divided by the outcome (did or did not become pregnant)

		Became pregnant (*n* = 205)	Did not become pregnant (*n* = 51)	*p*-Value
Age, median (IQR)		28 (7.50)	31 (7.00)	**0.043**^d^
CRP (mg/L), median (IQR)		0.78 (1.29)	0.95 (1.72)	**0.039**^d^
No. of cigarettes per day, median (IQR)		0 (0)	0 [1]	**0.015**^d^
Saliva secretion (mL/2 min), median (IQR)		2.40 (1.45)	2.00 (1.30)	0.134^d^
No. of teeth, median (IQR)		28 (1)	28 (1)	0.331^d^
No. of caries teeth, median (IQR)		0 (1)	0 (1)	0.160^d^
No. of periodontal pockets (if pockets), median (IQR)^a^		3 (5)	2 (5)	0.230^d^
VP (%)		10.70 (14.70)	10.40 (12.50)	0.118^d^
BOP (%)		10.71 (14.42)	8.64 (13.74)	0.544^d^
Diabetes, *n* (%)		0 (0)	0 (0)	–
Hormonal medication or infertility treatment, *n* (%)		11 (5.5)	5 (10.2)	0.233^e^
Has delivered previously, *n* (%)		74 (37.2)	11 (22.4)	0.052^e^
Education level, *n* (%)^b^	Low	53 (26.8)	19 (39.6)	0.068^e^
	Medium	87 (43.9)	22 (45.8)	
	High	58 (29.3)	7 (14.6)	
Current smokers, *n* (%)		28 (13.7)	14 (27.5)	**0.017**^e^
Has smoked ≥6 years, *n* (%)		22 (10.7)	13 (25.5)	**0.006**^e^
Smokes ≥6 cigarettes per day, *n* (%)		17 (8.3)	10 (19.6)	**0.019**^e^
BV positive, *n* (%)		41 (20.0)	14 (15.7)	0.246^e^
PAL, *n* (%)		24 (11.7)	8 (15.7)	0.442^e^
Periodontal pockets, *n* (%)^c^		38 (18.5)	7 (13.7)	0.419^e^
BOP ≥15%, *n* (%)		66 (32.2)	16 (31.4)	0.910^e^

*p*-Values <0.05 are considered significant (shown in bold).

^a^Periodontal pockets of ≥4 mm.

^b^Education level: low – junior high school or vocational school; medium – college, polytechnic, or a higher vocational institute; or high – academic university. Ten students were excluded because their education level was not known.

^c^More than one periodontal pocket of ≥4 mm.

^d^Non-parametric Mann–Whitney *U* two independent samples test.

^e^Chi-square test.

IQR, interquartile range; CRP, C-reactive protein; VP, visible plaque measured from six sites of teeth; BOP, bleeding on probing measured from six sites of teeth; BV, bacterial vaginosis; PAL, periodontal attachment loss of ≥1 mm.

All antibody levels measured from saliva and serum were higher among those with clinical PAL, and these differences were statistically significant for saliva (*p* = 0.001) and serum (*p* = 0.042) *A. actinomycetemcomitans* IgG, as well as saliva *P. gingivalis* IgA (*p* = 0.025) and IgG (*p* < 0.001). The PCR positivity for either bacterium did not significantly associate with clinical PAL (*p* = 0.2 for both). Both serum and saliva antibody levels were higher among subjects who were PCR positive for the corresponding bacterium compared to those who were PCR negative. For *P. gingivalis*, the differences were statistically significant for serum IgA (*p* < 0.001), serum IgG (*p* = 0.023), and saliva IgG (*p* < 0.001). For *A. actinomycetemcomitans*, the differences were significant for serum IgA (*p* = 0.009), serum IgG (*p* = 0.006), and saliva IgG (*p* < 0.001). The corresponding serum and saliva antibody levels had a moderate correlation with each other, with correlation coefficients ranging between 0.156 (*p* = 0.013) and 0.650 (*p* < 0.001).

Table 2 shows that *P. gingivalis* was significantly (*p* = 0.032) more frequently detected in saliva among those who did not become pregnant (8.3%) than among those who became pregnant (2.1%), and the median levels of salivary *P. gingivalis* IgA (*p* = 0.006) and IgG (*p* = 0.007) antibodies were higher among those who did not become pregnant compared to those who became pregnant. No significant differences were found between IgA or IgG serum concentrations (Table 2) or seropositivities to these two periodontal pathogens (data not shown) between these groups of women.

**Table 2.** Detection frequency of selected periodontal pathogens in saliva and antibody concentrations to these species in serum and saliva of study subjects (*n* = 256) as a comparison between those with success or no success in becoming pregnant

	Became pregnant (*n* = 205)	Did not become pregnant (*n* = 51)	*p*-Value
Saliva PCR, *n* (%)			
Pg positive	4 (2.1)	4 (8.3)	**0.032**^a^
Aa positive	9 (4.7)	1 (2.1)	0.416^a^
			
			
Antibody level, median (IQR)			
Serum (EU)			
Pg-IgA	0.31 (0.22)	0.35 (0.30)	0.324^b^
Pg-IgG	2.86 (1.63)	3.02 (2.20)	0.494^b^
Aa-IgA	0.49 (0.46)	0.44 (0.44)	0.920^b^
Aa-IgG	1.43 (1.48)	1.67 (1.99)	0.055^b^
Saliva (AU)			
Pg-IgA	0.86 (0.55)	1.07 (0.64)	**0.006**^b^
Pg-IgG	0.11 (0.13)	0.17 (0.22)	**0.007**^b^
Aa-IgA	0.93 (0.84)	0.99 (0.77)	0.628^b^
Aa-IgG	0.25 (0.32)	0.33 (0.51)	0.114^b^

*p*-Values <0.05 are considered significant (shown in bold).

^a^Chi-square test.

^b^Non-parametric Mann–Whitney *U* two independent samples test.

PCR, polymerase chain reaction; Pg, *Porphyromonas gingivalis*; Aa, *Aggregatibacter actinomycetemcomitans*; EU, ELISA units; AU, absorbance units.

Cox regression analysis confirmed that elevated salivary antibodies against *P. gingivalis* were associated with no success in conception (Table 3) and that this finding was independent of age, current smoking, socioeconomic status, BV, previous deliveries, and clinical PAL. The six women who were at the same time PCR positive for *P. gingivalis* and who belonged to the highest tertile of either *P. gingivalis* IgA or IgG (*n* = 96) had a HR of 3.75 (CI 1.01–13.9; *p* = 0.048) for not becoming pregnant compared to *P. gingivalis*–negative subjects with lower levels of salivary antibodies. In women who had more than one deepened periodontal pocket, serum *P. gingivalis* IgA associated with no success in conception with a HR of 1.62 (CI 1.03–2.54; *p* = 0.035). In addition, in the full Cox regression model, women who were older (*p* = 0.043), current smokers (*p* = 0.017), had smoked at least for 6 years (*p* = 0.006), smoked more cigarettes per day (*p* = 0.015), were of lower socioeconomic status (*p* = 0.027), had not delivered earlier (*p* = 0.043), or had higher CRP (*p* = 0.039) were more likely not to get pregnant. BV, PAL, the numbers of teeth or gingival pockets, or gingival bleeding did not differ between the groups. Salivary presence of or salivary antibodies against *A. actinomycetemcomitans* or serum antibodies against *P. gingivalis* or *A. actinomycetemcomitans* were not significantly associated with conception outcome.

**Table 3.** Associations of selected periodontal pathogens in saliva and antibody concentrations to these species in serum and saliva with delayed conception during a 12-month follow-up

	HR (95% CI), *p*-value
Saliva PCR	
Pg positive	**2.85 (1.00–8.24), 0.048**
Aa positive	0.56 (0.08–4.09), 0.565
	
Antibody level	
Serum (EU)	
Pg-IgA	1.16 (0.83–1.63), 0.385
Pg-IgG	1.00 (0.93–1.07), 0.950
Aa-IgA	1.22 (0.70–2.13), 0.485
Aa-IgG	1.12 (0.93–1.34), 0.226
Saliva (AU)	
Pg-IgA	**11.0 (3.01–40), <0.001**
Pg-IgG	**12.1 (2.63–55), 0.001**
Aa-IgA	1.82 (0.54–6.14), 0.331
Aa-IgG	2.58 (0.80–8.28), 0.111

*p*-Values <0.05 are considered significant (shown in bold).

The Cox regression models are adjusted for age, current smoking, socioeconomic status, bacterial vaginosis, previous deliveries, and clinical periodontal attachment loss.

HR, hazard ratio; CI, confidence interval.

## Discussion

This is the first report to demonstrate with microbiological and serological methods that a common periodontal pathogen associates with conception. The main finding was that the detection of *P. gingivalis* in saliva and elevated concentrations of salivary antibodies against this periodontal species significantly increase the risk for unsuccessful conception among young women. This result was independent of age, current smoking, socioeconomic status, BV, previous deliveries, and clinical PAL in Cox regression analysis. Periodontitis and infertility share some common risk factors such as age, smoking, low socioeconomic status, and obesity. In addition to these traditional risks and confounding factors, the results suggest that *P. gingivalis* may play a role in delayed conception or at least be a marker of this association.

Although both *P. gingivalis* and *A. actinomycetemcomitans* are considered etiological periodontal pathogens [17], their salivary presence did not differ between subjects with and without PAL in the present study. The detection frequencies of salivary *P. gingivalis* and *A. actinomycetemcomitans* in the Finnish adult population are 35.3% and 20.0% in the whole sample of subjects ≥30 years, and 13.0% and 16.0% in the lowest age group of 30–34 years, respectively [14]. The rates were markedly lower (3.1% and 3.9%) in the present study, probably reflecting the oral health of the healthy young women. Even though periodontal diseases may affect young adults, the bacterial counts are usually lower and the expression of the disease is generally milder than later in life. It has been shown previously that even the carriage and, further, the amount of the major periodontal bacteria is a stronger determinant of the systemic antibody response than the extent of periodontitis [18]. This most likely explains the present association between delayed pregnancy and salivary but not serum antibody response, although they moderately correlated with each other. Clinical periodontal characteristics such as PAL, the number of teeth or periodontal pockets, or amount of gingival bleeding did not differ between women who became pregnant and women with delayed conception. It is possible that salivary antibodies appear before serum antibodies, especially in subjects with no significant signs of periodontitis, as was the periodontal status of most of the study subjects. On the other hand, the women with signs of periodontitis and high serum IgA levels against *P. gingivalis* had an increased hazard of not becoming pregnant. Beck et al. have suggested that the quality and quantity of the host response to oral bacteria may be an exposure more relevant to systemic events than solely clinical periodontal measures [19]. The possible routes of systemic exposure to *P. gingivalis* are diverse, but they cannot be further clarified with the present study design.

Both *P. gingivalis* and *A. actinomycetemcomitans* are strongly associated with clinical periodontitis [17]. However, only the salivary presence of *P. gingivalis* was associated with delayed conception. The difference between *P. gingivalis* and *A. actinomycetemcomitans* may be due to the variation in specific virulence factors such as LPS, fimbriae, adhesins, and proteinases [20]. For example, the endotoxin activity of LPS may vary between Gram-negative species, depending on the structure of LPS [21]. Also, species-specific factors such as gingipains, cysteine proteinases, specifically produced by *P. gingivalis*, may exert immunomodulatory effects and therefore help the bacterium manipulate host response [20,22]. The association between *P. gingivalis* in saliva and fertility problems suggests that some specific bacterial features are involved, independent of the extent of clinical infection and inflammation. The same bacterial antigens that explain the association between *P. gingivalis* infection and adverse pregnancy outcomes in humans [23,24] and in animal models [25,26] may also delay conception. The underlying mechanism between *P. gingivalis* virulence and conception may also include more specific features, as recently shown with aspiration pneumonia by *P. gingivalis* in a mouse model: gingipains were crucial for the inflammatory response in the lungs, making a clear contribution to the development of pneumonia [27].

The strength of this study is that it a group of young Finnish women was recruited that was fairly homogenous considering socioeconomic status and general health. Limitations of this study are that information on the exact date of the discontinuation of contraception and how long it was being used before that was not available. It is not known if the subjects in need of periodontal therapy did actually receive treatment during the follow-up period. In addition, it was not studied further if the delayed conception was actually due to the presently studied women or their spouses. The distribution of infertility due to male factor is found to range from 20% up to 70% [28]. The association between *P. gingivalis* and delayed conception needs to be confirmed in other settings and a larger material, and the mechanisms explaining this association need to be clarified. The present data, however, encourage women in fertile age to maintain good oral hygiene and to attend periodontal evaluations regularly in order to avoid periodontal infection. Infertility is a major concern, and increasing healthcare resources are needed for infertility treatments. The dental and medical community should pay more attention to the common periodontal diseases potentially limiting conception or interfering with the success of becoming pregnant.
